# BCL2DB: database of BCL-2 family members and BH3-only proteins

**DOI:** 10.1093/database/bau013

**Published:** 2014-03-06

**Authors:** Valentine Rech de Laval, Gilbert Deléage, Abdel Aouacheria, Christophe Combet

**Affiliations:** ^1^Unité Bases Moléculaires et Structurales des Systèmes Infectieux, UMR 5086 CNRS - Université Claude Bernard Lyon 1, IBCP - 7, passage du Vercors, 69367 Lyon cedex 07, France and ^2^Molecular Biology of the Cell Laboratory, Ecole Normale Supérieure de Lyon, LBMC UMR 5239 CNRS – UCBL – HCL – ENS Lyon, 46 Allée d’Italie, 69364 Lyon Cedex 07, France

## Abstract

BCL2DB (http://bcl2db.ibcp.fr) is a database designed to integrate data on BCL-2 family members and BH3-only proteins. These proteins control the mitochondrial apoptotic pathway and probably many other cellular processes as well. This large protein group is formed by a family of pro-apoptotic and anti-apoptotic homologs that have phylogenetic relationships with BCL-2, and by a collection of evolutionarily and structurally unrelated proteins characterized by the presence of a region of local sequence similarity with BCL-2, termed the BH3 motif. BCL2DB is monthly built, thanks to an automated procedure relying on a set of homemade profile HMMs computed from seed reference sequences representative of the various BCL-2 homologs and BH3-only proteins. The BCL2DB entries integrate data from the Ensembl, Ensembl Genomes, European Nucleotide Archive and Protein Data Bank databases and are enriched with specific information like protein classification into orthology groups and distribution of BH motifs along the sequences. The Web interface allows for easy browsing of the site and fast access to data, as well as sequence analysis with generic and specific tools. BCL2DB provides a helpful and powerful tool to both ‘BCL-2-ologists’ and researchers working in the various fields of physiopathology.

**Database URL:** http://bcl2db.ibcp.fr

## Introduction

Two distinct groups of BCL-2–related proteins control the mitochondrial apoptotic pathway and probably other cellular processes as well (1, 2). The first group is formed by a family of homologs related to BCL-2 by a common ancestry, and the second group comprises a heterogeneous collection of evolutionarily and structurally unrelated proteins characterized by the presence of a single short stretch of sequence similarity with BCL-2, termed the BH3 motif.

BCL-2 homologous proteins share a similar α-helical bundle fold (the ‘BCL-2 domain’), have up to four different BH motifs (BH1-BH4) and can be either anti-apoptotic (e.g. BCL-2 and BCL-xL) or pro-apoptotic (e.g. Bax, Bak and Bid), whereas all of the BH3-only proteins are pro-apoptotic. Moreover, a variety of viral proteins have been found to be structurally similar to BCL-2 with or without obvious sequence similarity (3).

Since the discovery of the *bcl-2* gene 30 years ago, intense research in various disciplines has exponentially increased the quantity of data available on the BCL-2 family and BH3-only proteins. Therefore, it is of considerable interest to use bioinformatic tools to (i) understand the various groups of proteins structurally or functionally linked to BCL-2 and their implication in diseases; (ii) bring all the available information together in a specialized database [for which we have previously developed a prototype (4)]. We recently proposed a novel classification scheme for BCL-2–related proteins, based on phylogenetic information and computational analysis of sequence data (5, 6). Here, we describe an enhanced version of the BCL-2 database, a computer-annotated sequence database dedicated to BCL-2 homologous and BH3-only proteins, as well as the integrated Web interface that provides easy and efficient access to the data.

## The BCL2DB database

BCL2DB is available since July 2013. The release 2 comprises 1039 entries, including 880 BCL-2 homologous proteins (655 encoded by metazoan genomes and 225 from viruses) and 159 BH3-only proteins. Based on our new classification scheme, we built an automated workflow to feed BCL2DB. The workflow relies on a set of specific profile HMMs (7) derived from 40 reference protein sequences representative of the various orthologous subgroups present within the BCL-2–like and BH3-only groups. This computational pipeline was able to identify both close and distant homologs of BCL-2 (including viral members) as well as the known repertoire of BH3-only proteins when searching the UniProt Knowledgebase (UniProtKB) (8). The identified sequences are then annotated to provide entries in the European Nucleotide Archive (ENA) (9) EMBL-Bank format, which is loaded into a PostgreSQL relational database management system. Finally, sequence data sets are extracted and multiple sequence alignments are computed together with associated data. BCL2DB is updated on a monthly basis. All the programs of the computational pipeline have been written in Java, and SQL was used for database queries.

### Identification of BCL-2 homologous sequences and BH3-only sequences

**Figure 1.**
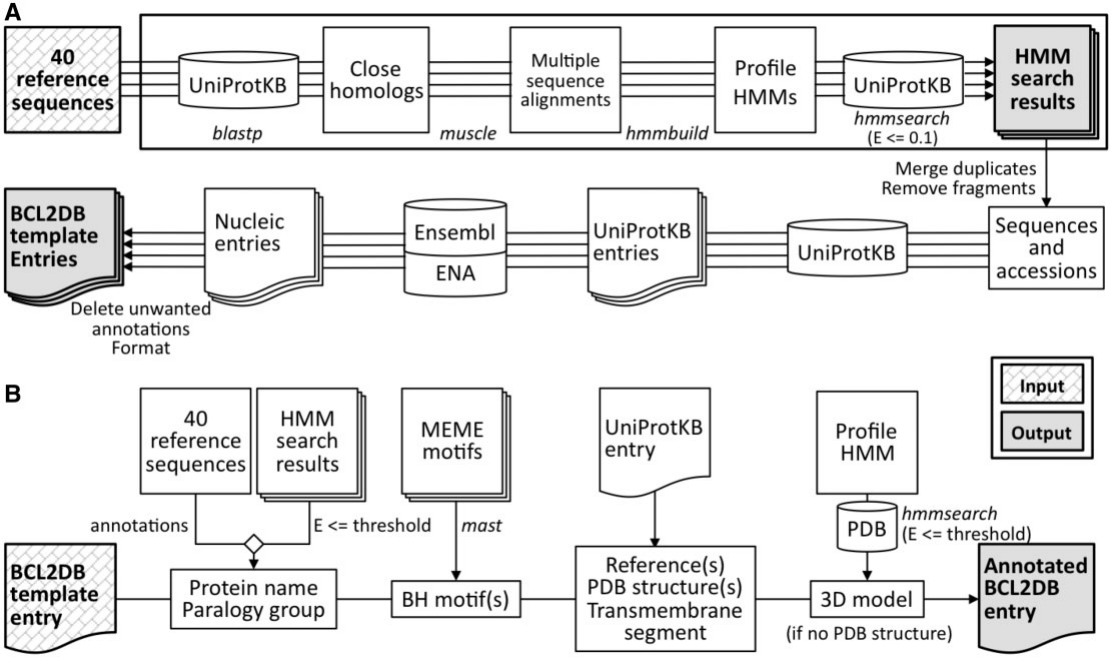
Description of the *FindBCL2* and *AnnotateBCL2* processes used to generate BCL2DB. External programs used by the processes are indicated in italics. (**A**) The upper part of the panel (boxed) describes the discovery mode of the *FindBCL2* program. The results are the profile HMMs and their associated classification E-value thresholds deduced after a HMM search against UniProtKB. The production mode used to generate the BCL2DB entry templates is described in the bottom part. After an *hmmsearch* on UniProtKB with the computed profile HMMs, the Ensembl or ENA entries are retrieved from cross-references or BLAST searches with nonfragment protein sequences and after removing duplicated sequences. Then, the entries are cleared of unwanted annotations and merged into a single one if they refer to the same Ensembl, Ensembl Genomes or ENA entry. (**B**) The *AnnotateBCL2* process enriches each BCL2DB entry template with annotations from reference sequences, sequence classification information (protein/gene name and orthology group/cluster), location of BH motifs and structural data retrieved from the PDB.

The *FindBCL2* program (Figure 1A) ensures sequence identification and provides two modes of execution: discovery and production. In the discovery mode, a profile HMM is computed (*hmmbuild* program of HMMER package 3.0) for each reference sequence (of individual BCL-2 homologs or BH3-only proteins) from a multiple alignment of their closest homologous sequences extracted after a BLAST search against UniProtKB with a score threshold tailored for each reference sequence. Each profile HMM is then used to search UniProtKB (*hmmsearch* program), and an E-value threshold is defined for use during the annotation process to classify the sequences into orthology groups (for BCL-2 homologous proteins) or clusters (for BH3-only proteins). The discovery mode is run periodically to improve the profiles sensitivity or when a new sequence is included in the seed set. The production mode is used to generate BCL2DB. The process starts by searching UniProtKB with the profile HMMs that were computed in the discovery mode. Then, for each selected sequence (E-value < 0.1) the Ensembl (10), Ensembl Genomes (11) or ENA entry is retrieved from UniProtKB cross-references or after a BLAST search. UniProtKB sequences corresponding to identical Ensembl or ENA entry are merged into one single entry. Unwanted annotations (*i.e*. uncertain, poor quality or nonconformity to the vocabulary standards) retrieved from Ensembl/ENA entries are then deleted to create a BCL2DB entry template that will be enriched with standardized data during the annotation procedure.

### Annotation procedure

The annotation procedure (*AnnotateBCL2* program; Figure 1B) starts from the entry templates generated for sequences that belong to the group of BCL-2 homologs or BH3-only proteins. The annotation process automatically affiliates each identified protein to its closest orthology group or cluster based on a specific curated gathering threshold cutoff (different for each profile). Above the threshold, the entry (typically a sequence from a nonmammalian organism) is considered as ‘unclassified’. Moreover, homemade BH1-4 motif profiles were developed (see below) for use in computational annotation of BCL2DB sequences to precise the positions of their respective BH region(s). Finally, the Protein Data Bank (PDB) (12) sequences are searched for known structures with the profile HMMs.

### BH motif annotation

We performed an *ab initio* motif discovery procedure by running the *meme* program of the MEME software suite (13) on a reference set of 158 amino acid sequences of BCL-2 homologous proteins and BH3-only proteins. Four position-specific scoring matrices corresponding to the four BH motifs were defined by *meme*. This original approach increases the sensitivity and specificity of BH-motif detection in protein sequences. The *mast* program uses the resulting position-specific scoring matrices to scan BCL2DB sequences for BH motifs. The *mast* results, which allow mapping of BH motifs onto BCL2DB sequences, are integrated in the BCL2DB entry as a protein sequence annotation.

### Entry content

The text format of a BCL2DB entry is an extension of the ENA EMBL-Bank format (14, 15). The BCL2DB accession numbers (*AC* line, repeated in *ID* line) are the UniProtKB accession numbers of the sequences identified by the profile HMM searches. In a BCL2DB Pearson/Fasta file, the accession number is associated with the gene/protein name and an isoform number (if needed) to compose the sequence identifier. The description *(DE* line) and keyword (*KW* line) fields of a BCL2DB entry contain information about the classification and the BH motifs of the sequence, as computed during the annotation procedure. The bibliographic references (*RN*, *RC*, *RP*, *RA*, *RT* and *RL* lines) are merged from the ENA and UniProtKB entries. Cross-references (*DR* lines and *db_xref* and *PRABI_prodft* qualifiers) to ENA, Ensembl, Ensembl Genomes, Gene Ontology (16), Human Protein Atlas (17), Protein Data Bank, RefSeq (18), NCBI Taxonomy (19) and UniProtKB are also provided. An additional cross-reference is provided to link each entry to the related BCL2DB reference sequence. The features (*FT* lines) retrieved from ENA, Ensembl and Ensembl Genomes entries are enriched in protein annotations through *PRABI_prodft* qualifiers under *mat_peptide* features. The *PRABI_prodft* qualifiers follow the feature table format of the UniProtKB database and describe information about proteins (e.g. chains, domains or sites). The protein annotation data added are the structural information and the presence of BH motifs. Known 3D structures, retrieved in *DR* lines of UniProtKB entries, or homologous sequences with an experimentally solved structure, retrieved by profile HMM searches on PDB sequences, are listed as *PRABI_prodft* of types PDB and MODEL3D, respectively. In both cases, information about PDB code, experimental technique used to solve the structure and the resolution in case of X-ray crystallography are provided. The BH motifs are listed as *PRABI_prodft* of type *MOTIF.*

## Web interface

BCL2DB is publicly accessible through a Web site, which was accessed 1556 times by 259 unique users since 26 July 2013 (representing 28 requests by day excluding internet robots and counting only access to data Web pages). The Web-based interface allows easy browsing of the site and fast access to data, thanks to the menu bar and the set of buttons available.

### BCL-2 menu

**Figure 2.**
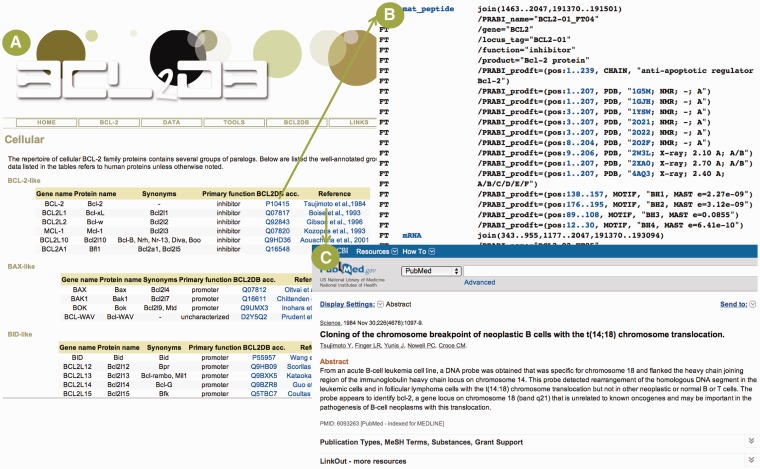
Example of general information available for BCL2DB reference sequences. (**A**) Cellular BCL-2 homologous reference sequences general information ordered by main clades (BCL-2-like, BAX-like and BID-like) and organized as tables. For each reference sequence, two links are provided to view the BCL2DB entry and the PubMed entry of the article describing the protein discovery. (**B**) Partial view of the feature table corresponding to the BCL2DB entry P10415. Links are provided to retrieve nucleotide (e.g. mRNA or mat_peptide) and protein sequences (e.g. PRABI_prodft), as well as to view entries of cross-referenced database (e.g. PDB or GO). (**C**) PubMed entry of the article by Tsujimoto *et al.* reporting the discovery of the *BCL-2* gene.

The *Nomenclature* submenu describes the terminology and classification used in BCL2DB with three protein subgroups (BCL-2 homologous, BH3-only proteins and structurally related proteins) composing the BCL-2 protein group. The *Domain* and *Motif* submenus list the clades, orthology groups and reference sequences existing within the three subgroups. For each reference sequence, the recommended protein and gene names, their synonyms, their primary function with regard to apoptosis, their accession number in BCL2DB and a literature citation with a link to PubMed are provided in a table (Figure 2). The *Community* submenu offers links to other Web resources related to cell death and apoptosis, as well as pointers to the community working in the field.

### Data menu

**Figure 3.**
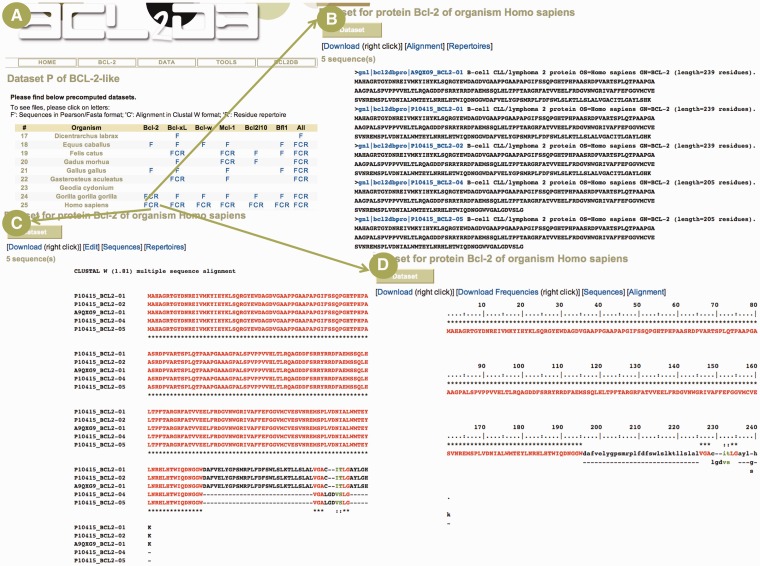
Example of a protein sequence data set. (**A**) Partial view of the page giving access to cellular BCL-2 homologous protein data sets. The table lists available data sets for the diverse species and proteins in the BCL-2-like clade. The user can access sequences in Fasta/Pearson format (F letter), multiple sequence alignment in Clustal W format (C letter) and residue repertoire (R letter). (**B**) The Fasta/Pearson file for *Homo sapiens* BCL-2 protein sequences. The sequence identifiers are built with the primary accession number, the protein name and an isoform number. A link is provided on sequence identifier to view the BCL2DB entry (Figure 2B). (**C**) The multiple sequence alignment computed with MUSCLE and displayed in Clustal W format. The color code used is red, green, black for residues that are conserved, strongly similar, weakly similar and variable in the alignment column, respectively, as defined by Clustal W. Dashes indicate gaps. (**D**) Residue repertoire computed from the previous alignment with the same color code.

The *DATA* menu provides access to sequence and structure data updated at the time of the database release computation by means of tables available in the Web pages. The tables allow simple and fast access to the data by biologists, avoiding them to build and execute complex dynamic SQL queries. For the sequence data sets, the table cells can contain F, C and R letters that provide links to Fasta files, color-coded multiple sequence alignments computed by means of MUSCLE (20) in Clustal W format and residue repertoires (Figure 3), respectively. A *frequencies* file is provided along with the repertoire that includes the Shannon entropy (21), a useful parameter to analyze conserved/variable alignment positions, and residue frequencies used to compute the repertoire and entropy. Furthermore, the alignments can be interactively edited with the ‘EditAlignment’ applet developed by our team. The full-length sequence data sets are ordered according to BCL2DB classification scheme, orthology groups, species and gene/protein recommended names. The motif sequence data sets are ordered by protein recommended names and BH motifs. The *All* rows and columns give access to all sequences of a given species, gene/protein or BH motif. The structure data sets follow the BCL2DB classification scheme, and the tables provide for a given protein the structures available in the PDB with links to download and view the structure file, the experimental method used to solve the structure, including the resolution for X-ray crystallography, the deposition year, the source organism and the reference with a link to PubMed.

### Tools menu

**Figure 4.**
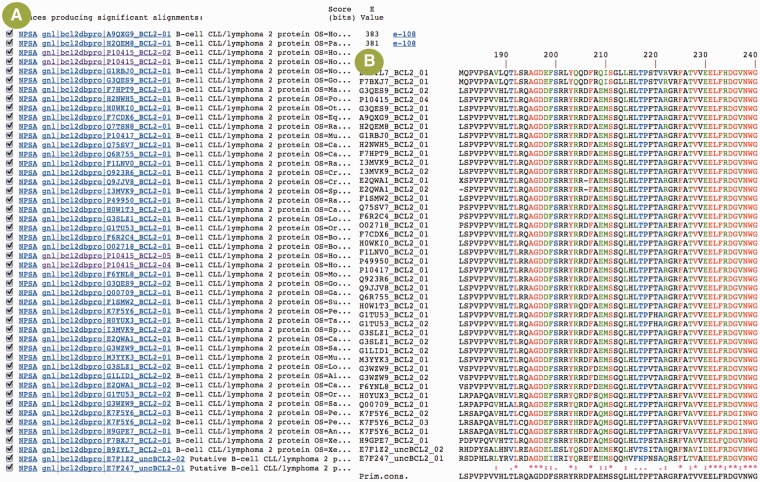
Example of sequence analysis with BCL2DB sequences and the NPS@ server. (**A**) Results of a blastp search with the BCL-2 protein sequence (P10415) against the BCL2DB protein sequences. A first link (NPSA) is provided to extract the matching sequence from the sequence databank and perform further analyses with the set of tools available in NPS@. The second link on the sequence identifier is provided to view the BCL2DB entry (Figure 2B). The pairwise sequence alignment between the query sequence and the matching sequence can be viewed, thanks to the link on the E-value. (**B**) Partial view of the multiple sequence alignment of BCL2DB BCL-2 proteins in the region of the BH3 motif. The alignment was computed with sequences selected and extracted from the blastp results. The color code used is red, green, black for residues that are conserved, strongly similar, weakly similar and variable in the alignment column, respectively, as defined by Clustal W. Dashes indicate gaps.

**Figure 5.**
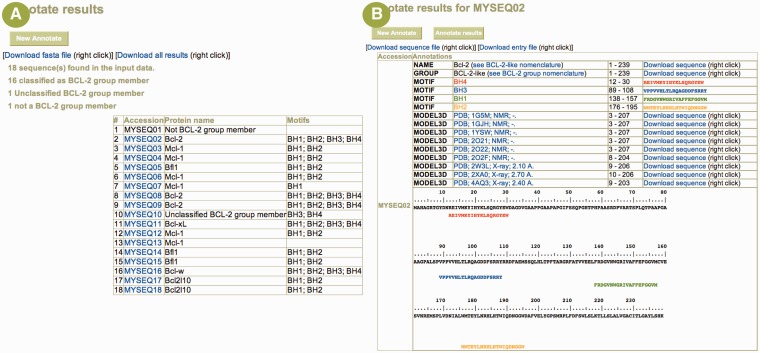
Example of user sequence annotation with the *Annotate* tool. (**A**) The main result page summarizes the submitted data and offers a table listing each input sequence (here, 18 sequences were uploaded) with its predicted protein name and its BH motif composition. A link to a detailed result page is provided on each sequence identifier when the sequence is annotated as belonging to the BCL-2 protein group. (**B**) The detailed result page for sequence *MySeq02* displays the predicted protein name, the classification, the BH motif composition and the homologous known 3D structures. Numerous links allow the user to (i) download the sequences corresponding to the various annotations, (ii) download a UniProtKB formatted entry of the annotated sequence and (iii) browse structure entry at the PDB Web site. The submitted sequence is also displayed with colored BH motifs for easy cut-and-paste to other programs.

The analysis tools provided with the database are categorized either as generic or specialized. The generic analysis tools are available through the NPS@ server (22), our integrated resource for sequence analysis. For instance, BCL2DB nucleotide and protein sequences can be searched with BLAST (23) and selected sequences can be extracted and aligned with Clustal W (24) (Figure 4). The *Annotate* specialized tool permits users to annotate their own protein sequences with the set of programs used to feed BCL2DB. Users can determine whether their sequence is predicted as belonging to either the BCL-2 homologous or BH3-only group, its classification according to the orthology groups or clusters and its BH motif arrangement. The *Annotate* main result page contains a summary table showing each input sequence listed with its classification, its name and a link to access the detailed result page (Figure 5). Information displayed in the latter page includes classification, sequence name and the protein annotations as described in the *Entry Content* paragraph.

### BCL2DB menu

General information about the database is provided under this menu. The users have access to (i) the composition of the scientific advisory board (*About* submenu), (ii) a contact form to send messages to the BCL2DB team, (iii) the help about the Web interface, (iv) the news related to BCL2DB releases and changes, as well as Web site updates, and (v) the usage statistics.

## Conclusion and perspectives

BCL2DB is a collection of computer-annotated BCL-2–related sequences. The automatic annotation process used to generate the BCL2DB entries guarantees updates of the data and standardized annotations. The latter allow efficient keyword searches useful to generate sequence data sets available through the Web interface. Sequences can be retrieved for further analysis with a set of bioinformatics tools available in the NPS@ server. The BCL2DB Web site also allows researchers to access up-to-date knowledge about BCL-2 family members and BH3-only proteins and to annotate their own sequences through the BCL2DB automatic annotation process. In its current implementation, BLC2DB offers a good template to integrate new annotation data that will enrich its content in the future (*e.g.* gene expression, interaction data, information on posttranslational modifications) and will enhance its Web site with new analysis tools (*e.g.* to identify novel BH3-only proteins and splice variants) and a search tool to perform dynamic queries on the database to extract data sets of interest to the user. BCL2DB can serve as a reference for the analysis of data generated by means of high-throughput technologies. We put a lot of attention and rigor in developing BCL2DB to provide a helpful and powerful tool to both ‘BCL-2-ologists’ and researchers working in the various fields of physiopathology.
